# A mathematical model and CD4+ lymphocyte dynamics in HIV infection.

**DOI:** 10.3201/eid0204.960405

**Published:** 1996

**Authors:** T Hraba, J Dolezal

**Affiliations:** Institute of Molecular Genetics, Prague, Czech Republic. tomas.hraba@img.cas.cz

## Abstract

The paper presents a model of CD4 + lymphocyte dynamics in HIV-infected persons. The model incorporates a feedback mechanism regulating the production of T lymphocytes and simulates the dynamics of CD8 + lymphocytes, whose production is assumed to be closely linked to that of CD4 + cells. Because CD4 + lymphocyte counts are a good prognostic indicator of HIV infection, the model was used to simulate such therapeutic interventions as chemotherapy and active and passive immunization. The model also simulated the therapeutic administration of anti-CD8 antibodies; this intervention was assumed to activate T-cell production by activating a feedback mechanism blocked by the high numbers of CD8 + lymphocytes present in HIV-infected persons. The character and implications of the model are discussed in the context of other mathematical models used in HIV infection.


### A Mathematical Model and CD4+ Lymphocyte Dynamics in HIV Infection

**Tomáš Hraba* and Jaroslav Doležal†**
*Institute of Molecular Genetics, Prague, Czech Republic, †Honeywell Technology Center, Prague, and Institute of Information Theory and Automation, Prague, Czech Republic

The paper presents a model of CD4+ lymphocyte dynamics in HIV-infected persons. The model incorporates a feedback mechanism regulating the production of T lymphocytes and simulates the dynamics of CD8+ lymphocytes, whose production is assumed to be closely linked to that of CD4+ cells. Because CD4+ lymphocyte counts are a good prognostic indicator of HIV infection, the model was used to simulate such therapeutic interventions as chemotherapy and active and passive immunization. The model also simulated the therapeutic administration of anti-CD8 antibodies; this intervention was assumed to activate T-cell production by activating a feedback mechanism blocked by the high numbers of CD8+ lymphocytes present in HIV-infected persons. The character and implications of the model are discussed in the context of other mathematical models used in HIV infection.

The increased efficiency of modern computer techniques has expanded the possibilities of mathematical modeling in an unprecedented way. However, medical and biologic research has not taken full advantage of these possibilities. Mathematical models are, in fact, working hypotheses that require clear formulation and quantitative definition of factors and relations included in the model. These requirements may discourage biologic and medical research scientists from using mathematical models because quantitative data are often not available to them, or are available only to a limited extent. However, current computer techniques offer the possibility of quickly testing different estimates of a realistic, probable choice. The increased efforts required to construct mathematical models are amply rewarded by the quantitative predictions generated by the models.

In the absence of adequate animal models, mathematical modeling of HIV infection is especially important. Many adequate models of this infection have been formulated (e.g., 1-3), but they have had a relatively small impact on clinical and experimental research. Mathematical modeling, however, was an integral and important part of evaluating recently obtained data on HIV turnover in infected persons (4,5).

### The Mathematical Model

We had simulated the dynamics of lymphocytes in immunologic tolerance (6) for a decade when we became interested in modeling lymphocyte dynamics in HIV-infected persons (7,8). The tolerance model simulates escape from tolerance of a nonreplicating protein antigen and is based on the assumption that lymphocytes specific to the tolerated antigen start to appear when the concentration of the tolerated antigen drops below the threshold level required for tolerance induction in differentiating new lymphocytes. Our model of HIV infection concentrated on CD4+ lymphocytes because the depletion of this T-cell subpopulation, and the parallel decrease in the helper activity of T lymphocytes, seemed to be the major immune system defect caused by HIV infection. When we started to construct the model, the widely held view was that the decrease of this T-cell subpopulation is not caused by the cytopathic effect of the virus

Address for correspondence: Tomáš Hraba, Institute of Molecular Genetics, Flemingovo n. 2, 16637 Prague, Czech Republic; fax: 42-2-2431-0955; e-mail: tomas.hraba@img.cas.cz.

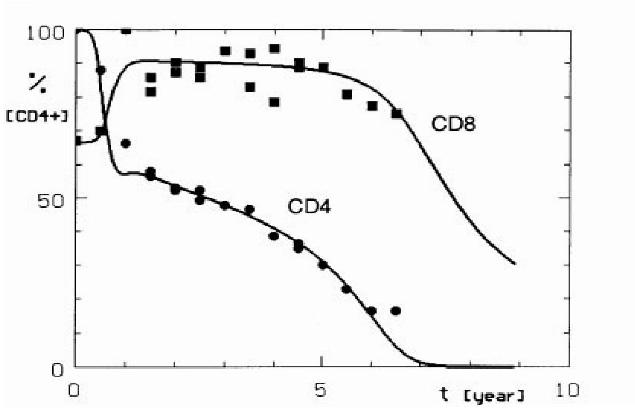

Figure 1.* Simulated CD4$^+$ and CD8$^+$ lymphocyte dynamics in HIV infection compared with observed mean T-cell values for CD4$^+$ lymphocytes (circles) and CD8$^+$ lymphocytes (squares).

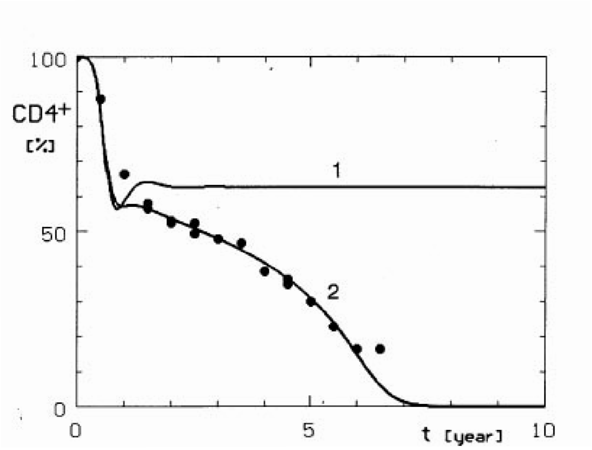

Figure 2.* Comparison of only linear (curve 1, $\nu$ =1.0) T-helper activity decrease with nonlinear (standard curve 2, $\nu$ = 1.6).

*CD4$^+$ cell observed values are depicted as circles and those of CD8$^+$ lymphocytes as squares. Both simulated and observed values are depicted as a percentage of normal CD4$^+$ lymphocyte numbers (the normal value of CD8$^+$ lymphocytes is thus 66.7%).

because in infected persons too few CD4$^+$ cells expressed HIV. A direct or indirect effect of HIV products on these cells was, therefore, considered to cause this depletion (9).

In both immunologic tolerance and HIV infection, antigen seemed to eliminate lymphocytes: in immunologic tolerance, lymphocytes carrying the specific antigen receptor were affected, and in HIV infection, the entire CD4$^+$ lymphocyte population was the target of HIV products. The dynamics of the affected lymphocyte pools were mutually inverse: in tolerance, the number of specific lymphocytes increased with time because the antigen concentration decreased and in HIV infection, the CD4$^+$ lymphocyte count decreased because of the rising level of HIV products as the infection progressed.

In the model, CD4$^+$ cell depletion was assumed to result, directly or indirectly, from an effect of HIV products, where HIV proliferated at the same rate during the whole course of the infection; therefore, once a simulated substantial decline of CD4$^+$ lymphocytes started, it progressed rapidly to their total depletion. However, previous studies indicated that an early decline in these cells occurs shortly after infection, is followed by a period of slow decline, and then the decline accelerates again about the time AIDS develops (10,11). However, if we assume that HIV proliferation is limited by a helper T-cell-dependent immune reaction, our model could simulate all the observed phases of CD4$^+$ cell dynamics well (8,12). In Figure 1, simulation curves of CD4$^+$ and CD8$^+$ lymphocytes (Appendix) are compared with the mean observed values of these cells in HIV-infected persons (10,12).

The immune reaction limiting HIV proliferation, postulated in the model, was assumed to be a specific cytotoxic activity of T cells that required the cooperation of helper T cells. However, the model is not dependent on this assumption, and any T helper-cell-dependent immune reaction can play this role. Other mechanisms are possible candidates for this function. Recently, interest was focused on cytokines produced by CD8$^+$ lymphocytes that inhibit HIV proliferation (13,14), an effect discovered much earlier (15,16). As far as this activity depends on T-helper cells, this situation can be simulated by our model. If T-helper cells did not play any substantial role in this mechanism, our model would not be applicable.

In our model, CD4$^+$ lymphocyte dynamics were successfully simulated only if T-helper-cell activity did not decrease linearly with the decline of CD4$^+$ lymphocytes but faster than these T cells. This relation is expressed by the power coefficient $\nu$ in Equation 6

(Appendix), which must have a value >1.0 (7,8). If the decrease of helper activity was assumed to be directly proportional to the number of CD4$^+$ lymphocytes ($\nu$ = 1.0), it was possible to simulate only the initial phase of the CD4$^+$ dynamics—the early drop of these cells. Then a permanent steady state of the CD4$^+$ cell level was established (Figure 2, curve 1). This finding could be of interest for elucidating mechanisms involved in the long-term, and possibly permanent, survival of some HIV-infected persons (17-19) whose condition seems to be characterized by an equilibrium between HIV infection and a protective immune reaction. Although some of these persons have CD4$^+$ lymphocyte numbers in the normal range, their cases do not necessarily contradict the model's prediction of a stabilized HIV infection because with a small HIV load the steady-state CD4$^+$ cell numbers might be indistinguishable from normal ones, given the broad range of normal values.

We are not able to ascribe a definite mechanism to the necessary assumption that the activity of T-helper cells declines faster than the number of CD4$^+$ lymphocytes. The faster decline could be caused by the disruption of the lymphoid tissue structure by HIV infection (20). Another possible cause is the increasing HIV variation as infection progresses (21), if this variation led to decreased sensitivity, or even resistance, to the protective immune reaction of at least a part of the virus population.

From our model of immunologic tolerance (6), two compartments of the studied lymphocytes were retained in the model of HIV infection (7): mature and immature CD4$^+$ cells. We incorporated immature lymphocytes in the model of immunologic tolerance because they were more sensitive to tolerance induction. The assumption of immature CD4$^+$ lymphocyte sensitivity to elimination by HIV did not influence the simulation results substantially (22); therefore, only mature CD4$^+$ cells were considered to be eliminated by the effect of HIV in most simulations we carried out, including those described in this article.

Different mechanisms of CD4$^+$ lymphocyte depletion caused by HIV infection were simulated by various modifications of our model, which assumed either a direct or indirect effect of HIV (22). Because no substantial difference was observed in simulation results, for convenience reasons, most of the work, including the examples presented in this article, assumed that cytotoxic cells limiting HIV proliferation are also instrumental in depleting CD4$^+$ lymphocytes by eliminating those have HIV products. This assumption seems to be supported by recent clinical findings (4,5).

Adleman suggested that the depletion of CD4$^+$ lymphocytes might activate some homeostatic mechanism that increases their production (24). He assumed that this homeostatic mechanism increased production of both CD4$^+$ and CD8$^+$ lymphocytes and did not discriminate between the two T-cell subpopulations.

Adleman also suggested that the substantial and permanent depletion of CD4$^+$ lymphocytes in HIV-infected persons might activate this mechanism. However, because only CD4$^+$ cells are destroyed in HIV infection, the newly produced CD8$^+$ lymphocytes would accumulate. An increase in the number of CD8$^+$ lymphocytes was actually observed in HIV-infected persons, while the total number of T cells remained in the normal range. The increase in the CD8$^+$ cell count might switch off the homeostatic mechanism that increases T-cell production, and as a consequence, cause or at least aggravate the CD4$^+$ lymphocyte depletion (24,25). Because this view was supported by convincing evidence, we incorporated this feedback mechanism in our model (22). When we compared quantitatively the simulated CD8$^+$ cell increase with the observed values, they did not agree well, especially in later phases of the infection when the simulated values continued to increase, while the observed CD8$^+$ lymphocyte counts started to decline. When it was assumed that HIV infection constrained the influx of both CD4$^+$ and CD8$^+$ lymphocytes, satisfactory simulation results were obtained (26) (Figure 1).

### Modeling Therapeutic Interventions

Because CD4$^+$ lymphocyte counts are a good prognostic indicator of HIV infection (27), our model is suitable for simulating different therapeutic interventions. The

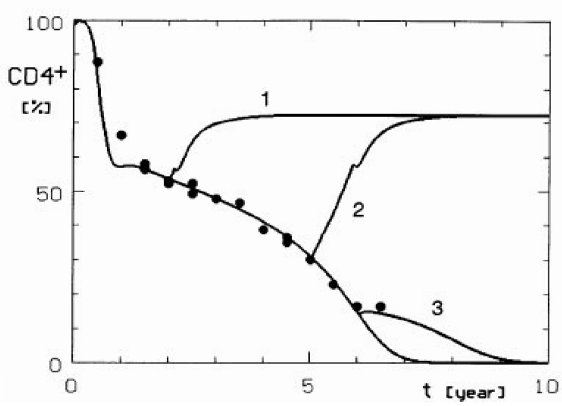

Figure 3.* Simulated effect of permanent AZT treatment (ξ = 0.005) started 2m 5m or 6 years after the acquisition of HIV infection—curves 1, 2, and 3, respectively.

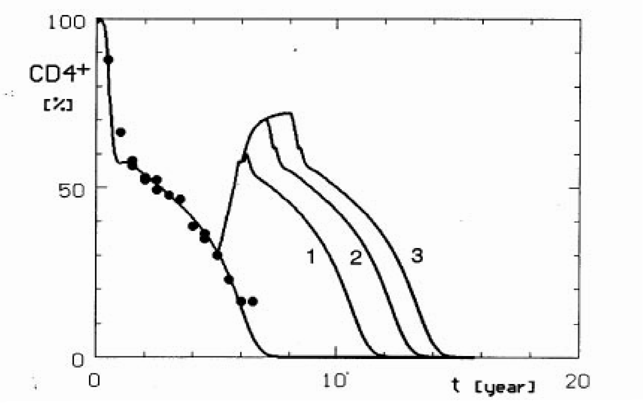

Figure 4.* Simulated effect of temporary AZT treatment (ξ = 0.005) started 5 years after the acquisition of HIV infection and lasted 1, 2, or 3 years—curves 1, 2, and 3, respectively.

*$CD4^+$ cell observed values are depicted as circles and those of $CD8^+$ lymphocytes as squares. Both simulated and observed values are depicted as a percentage of normal $CD4^+$ lymphocyte numbers (the normal value of $CD8^+$ lymphocytes is thus 66.7%).

model has been used to simulate zidovudine (AZT) chemotherapy and specific immunotherapy, both active and passive (28-30). The intensity of most therapies we simulated did not completely eradicate the infection, a situation common to the treatments now available. If a therapy that is assumed not to eradicate HIV infection is simulated to be administered permanently and to retain undiminished effectiveness, the observed overall result (besides slight differences in the dynamics of the changes induced by the various therapeutic measures) is the establishment of a new steady-state level of $CD4^+$ lymphocytes (Figure 3). The height of this level reflects the effectiveness of the therapy: the nearer to normal values, the more effective the interventions. This steady state is always lower than a normal state. Even when the therapy is started at a later stage of HIV infection, the obtained steady state is the same; its value depends only on the effectiveness of the therapy, regardless of the onset of treatment (Figure 3, curves 1 and 2).

However, this result is valid up to a certain point only: when the $CD4^+$ lymphocyte numbers are too low, therapy can no longer reverse the $CD4^+$ cell depletion; it can only slow the decrease and does not establish a steady state (Figure 3, curve 3). The stage of infection in which depletion cannot be reversed varies with different therapies and with different intensities of the same therapy (e.g., AZT doses). It occurs earlier in the course of the infection with less effective treatments than with more effective ones. Actually, there is a point in the intensity of each treatment when it is possible to stop further $CD4^+$ cell decline and establish a steady state with $CD4^+$ cell numbers corresponding to their value at the onset of therapy.

Another aspect of the therapies we simulated was their temporary application. If the therapy eliminated the virus completely, $CD4^+$ lymphocyte counts returned to normal for good. Our simulations concentrated mainly on therapies leading only to limitation of the viral load, and in consequence, to an increase in the numbers of $CD4^+$ cells. When the treatment was stopped, the decline of $CD4^+$ cells started again and essentially proceeded at the same rate as in untreated persons (Figure 4). The maximal increase of $CD4^+$ lymphocytes obtained by such intervention corresponded to that induced by a permanent application of a treatment of the same intensity. Of course, if the applied treatment did not last long enough to allow the $CD4^+$ cells to reach that level, only a lower maximal value was obtained.

According to the results of our simulations temporary therapy only prolonged survival, although extended survival could

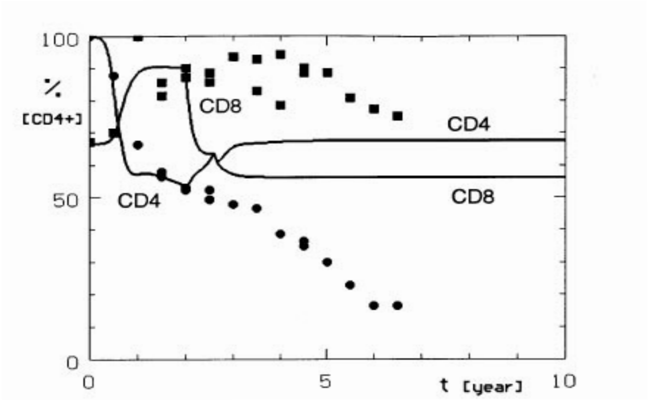

Figure 5.* Simulated CD4+ and CD8+ lymphocyte dynamics after permanent treatment with anti-CD8 antibodies started 2 years after the acquisition of the HIV infection. Cells mediating the protective anti-HIV immune reaction are not affected by this treatment ($\rho_R$ = 0.007, $\rho_C$ = 0.0).

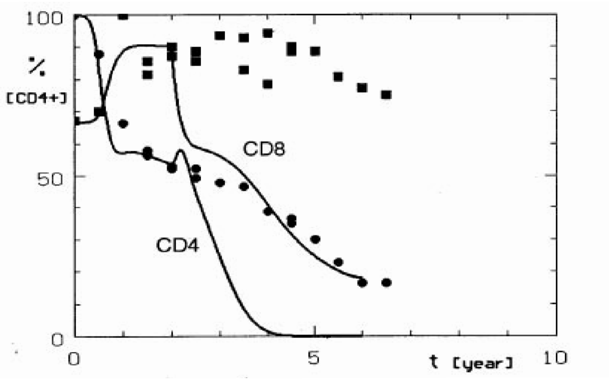

Figure 6.* Simulated CD4+ and CD8+ lymphocyte dynamics after permanent treatment with anti-CD8 antibodies started 2 years after the acquisition of the HIV infection. Cells mediating the protective anti-HIV immune reaction are also affected by this treatment ($\rho_R$ = 0.007, $\rho_C$ = 0.007).

*CD4+ cell observed values are depicted as circles and those of CD8+ lymphocytes as squares. Both simulated and observed values are depicted as a percentage of normal CD4+ lymphocyte numbers (the normal value of CD8+ lymphocytes is thus 66.7%).

exceed the length of treatment. This situation is relevant especially to chemotherapy that loses effectiveness after a relatively short time, probably because of drug resistance acquired by HIV. However, even immunotherapy cannot be expected to retain its undiminished effectiveness for a prolonged period.

### Therapeutic Depletion of CD8+ Lymphocytes

It has been suggested that lowering the number of CD8+ lymphocytes in HIV-infected persons by administering anti-CD8 antibodies could activate the homeostatic mechanism, thus increasing production of both CD4+ and CD8+ T cells (24,25). However, this mechanism might be blocked by the high numbers of CD8+ lymphocytes present in HIV infection, as discussed above. Therefore, these authors assumed that a depletion of CD8+ lymphocytes brought about by administering anti-CD8 antibodies might unblock this feedback mechanism and that this could counteract the depletion of CD4+ cells caused by HIV infection.

When this regulatory mechanism was incorporated in our model, we were able to simulate this situation (26,30). Reducing CD8+ lymphocyte values to numbers not much below normal stops the further decline in CD4+ cells and brings them to a steady state, which may be higher than their pretreatment number (Figure 5). Lower doses of anti-CD8 antibodies also stopped further decline of CD4+ cells, but the achieved steady-state level is lower than in the illustrated case. On the contrary, the CD4+ lymphocyte increase is larger with higher antibody doses. As in other cases of temporary treatment, the decrease in CD4+ values starts again after anti-CD8 antibody administration is discontinued.

However, such a therapeutic effect of CD8+ depletion is achieved only if lymphoid cells eliminated by the administered antibody are assumed not to participate in the anti-HIV immune reaction that limits virus proliferation. This is not the case with cytotoxic T cells, or with CD8+ lymphocytes responsible for the production of cytokines that inhibit HIV proliferation. This situation could also be simulated with our model. When these cells assumed to cause a protective immune reaction were also eliminated by the administered anti-CD8 antibody, the CD4+ lymphocyte decrease did not stop; it even accelerated (Figure 6). All doses of anti-CD8 antibody, even very small ones, increased the depletion of CD4+ lymphocytes under this assumption, although the deteriorating effect was not so strong when small doses of antibody were used.

For the sake of brevity, we refrained from presenting examples from other models, although in addition to providing some quantitative differences in simulated results, these examples could be of interest in modeling other aspects of HIV infection (31-33), and the comparison of other results with those of our model would add another dimension to this article. Different models, or some of their parts, can be combined, and in this way the actual situation might be simulated even better; on the other hand, a particular model might simulate a specific situation and help clarify some questions posed by clinical or experimental studies.

*Dr. Hraba is affiliated with the Institute of Molecular Genetics of the Academy of Sciences of the Czech Republic. His early research activities involved primarily the problem of immunologic tolerance. The work of Dr. Hraba is supported in part by grant A50502604 from the Academy of Sciences of the Czech Republic.*

*Dr. Doležal is a manager of Honeywell Technology Center, Prague. His research interests include theoretical and applied aspects of optimal control and mathematical programming and applications of mathematical modeling methods to dynamic systems of both technical and biologic origin. He has also coordinated large applied projects with industry.*

### References

1. Nowak MA, May RM. Coexistence and competition in HIV infection. J Theor Biol 1992;159:329-42.
2. Perelson AS, Kirschner DE, De Boer R. Dynamics of HIV infection of CD4+ T cells. Math Biosci 1993;114:81-125.
3. McLean AR. The balance of power between HIV and the immune system. Trends Microbiol 1993;1:9-13.
4. Ho DD, Neumann AU, Perelson AS, Chen W, Leonard JM, Markowitz M, et al. Rapid turnover of plasma virions and CD4 lymphocytes in HIV-1 infection. Nature 1995;373:123-6.
5. Wei X, Ghosh SK, Taylor ME, Johnson VA, Emini EA, Deutsch, P, et al. Viral dynamics in human immunodeficiency virus type 1 infection. Nature 1995;373:117-22.
6. Klein P, Hraba T, Doležal J. The use of immunological tolerance to investigate B lymphocyte replacement kinetics in chickens. J Math Biol 1983;16:131-40.
7. Hraba T, Doležal J. Mathematical model of CD4+ lymphocyte depletion in HIV infection. Folia Biol (Praha) 1989;35:156-63.
8. Hraba T, Doležal J. Contribution of mathematical modelling to elucidation of AIDS pathogenesis. J Biol Systems 1993;1:349-62.
9. Fauci AS. The human immunodeficiency virus: infectivity and mechanisms of pathogenesis. Science 1988;239:617-22.
10. Lang W, Perkins H, Anderson RE, Royce R, Jewell N, Winkelstein W, et al. Patterns of T lymphocyte changes with human immunodeficiency virus infection: from seroconversion to the development of AIDS. J AIDS 1989;2:63-9.
11. Margolick JB, Donnenberg AD, Muños A, Park LP, Bauer KD, Giorgi JV, et al. Changes in T and non-T lymphocyte subsets following seroconversion to HIV-1: stable CD3+ and declining CD3- populations suggest regulatory responses linked to loss of CD4 lymphocytes. J AIDS 1993;6:153-61.
12. Doležal J, Hraba T. Model-based analysis of CD4+ lymphocyte dynamics in HIV infected individuals. II. Evaluation of the model based on clinical observations. Immunobiology 1991;182:178-87.
13. Baier M, Werner A, Bannert N, Metzner K, Kurth R. HIV suppression by interleukin-16. Nature 1995;378:563.
14. Cocchi F, DeVico AL, Garzino-Demo A, Arya SK, Gallo RC, Lusso P. Identification of RANTES, MIP-1$\alpha$, and MIP-1$\beta$ as the major HIV-suppressive factors produced by CD8+ T cells. Science 1995;270:1811-5.
15. Walker CM, Moody DJ, Stites DP, Levy JA. CD8+lymphocytes can control HIV infection in vitro by suppressing virus replication. Science 1986;234:1563-6.
16. Mackewicz C, Levy JA. CD8+ cell anti-HIV activity: nonlytic suppression of virus replication. AIDS Res Hum Retrovir 1992;8:1039-50.
17. Muños A, Kirby AJ, He YD, Margolick JB, Visscher BR, Rinaldo CR, et al. Long-term survivors with HIV-1 infection: incubation period and longitudinal pattern of CD4+ lymphocytes. J AIDS 1995;8:496-505.
18. Cao Y, Qin L, Zhang L, Safrit J, Ho DD. Virologic and immunologic characterization of long-term survivors of human immunodeficiency virus type 1 infection. N Engl J Med 1995;332:201-8.
19. Pantaleo G, Menzo S, Vaccarezza M, Graziosi C, Cohen OJ, Demarest JR, et al. Studies in subjects with long-term nonprogressive human immunodeficiency virus infection. N Engl J Med 1995;332:209-16.
20. Frost SDW, McLean AR. Germinal centre destruction as a major pathway of HIV pathogenesis. J AIDS 1994;7:236-44.
21. Coffin JM. HIV population dynamics in vivo: implications for genetic variation, pathogenesis, and therapy. Science 1995;267:483-9
22. Hraba T, Doležal J, Čelikovský S. Model-based analysis of CD4+ lymphocyte dynamics in HIV infected individuals. Immunobiology 1990;181:108-18.
23. Zinkernagel RM, Hengartner H. T-cell-mediated immunopathology versus direct cytolysis by virus: implications for HIV and AIDS. Immunol Today 1994;15:262-8.
24. Adleman LM. On the maintenance of T cell populations. Technical Report. Los Angeles: Univ. of California: Dept. of Computer Science, 1988.

25. Adleman LM, Wofsy D. T-cell homeostasis: implications in HIV infection. J AIDS 1993;6:144-52.
26. Hraba T, Doležal J.A mathematical model of CD8+ lymphocyte dynamics in HIV infection. Folia Biol (Praha) 1995;41:304-18.
27. Stein DS, Korvick JA, Vermund SH. CD4+ lymphocytes cell enumeration for prediction of clinical course of human immunodeficiency virus disease: a review. J Infect Dis 1992;165:352-63.
28. Hraba T, Doležal J. Mathematical modelling of chemotherapy in HIV infection. Folia Biol (Praha) 1994;40:103-11.
29. Doležal J, Hraba T. Mathematical modelling of immunotherapy in HIV infection. Folia Biol (Praha) 1994;40:193-9.
30. Hraba T, Doležal J. Mathematical modelling of HIV infection therapy. Int J Immunopharmacol 1995;17:523-6.
31. De Boer JR, Boerlijst MC. Diversity and virulence thresholds in AIDS. Proc Natl Acad Sci USA 1994;94:544-8.
32. Essunger P, Perelson AS. Modeling HIV infection of CD4+ T-cell subpopulations. J Theor Biol 1994;170:367-91.
33. Nelson GW, Perelson AS. Modeling defective interfering virus therapy for AIDS: conditions for DIV survival. Math Biosci 1995;125:127-53.
34. Doležal J, Hraba T, Čelikovský S. Influence of the cell interaction parameters on the simulated CD4+ lymphocyte depletion in HIV infection. Folio Biol (Praha)1991:87:42-51.

### Appendix

The model considers immature and mature CD4+ ($\bar{P}$ and $P$ cells) and CD8+ lymphocytes ($\bar{R}$ and $R$ cells). As normal values of $R$ cells equal about two thirds of those of $P$ cells, it is assumed that normal $\bar{R}$ values correspond in a similar way to 2/3 of $\bar{P}$ cells. The sizes of these cell compartments at time $t$ are described by Eqs. (1)-(4). The amount of HIV products at time $t$ is given by Eq. (5). Finally, Eq. ( 6) gives the number of cytotoxic T cells specific for HIV ($C$ cells) at time $t$. In the model used, these cells both limit proliferation of HIV, as indicated in Eq. (5), and effect destruction of CD4+ cells presenting HIV products according to Eqs. (1)-(2).

$$\frac{d\bar{P}(t)}{dt} = \frac{I_P + f\left[\left(P_0 - P(t)\right) + \left(R_0 - R(t)\right)\right]}{d(t)} - \bar{\tau}_P\bar{P}(t) - \bar{c}_P a(t)C(t)\bar{P}(t), \quad \bar{P}(0) = \bar{P}_0 \qquad (1)$$

$$\frac{dP(t)}{dt} = \bar{\tau}_P\bar{P}(t) - \tau_P P(t) - c_P a(t)C(t)P(t), \qquad P(0) = P_0 \qquad (2)$$

$$\frac{d\bar{R}(t)}{dt} = \frac{2}{3}\,\frac{I_P + f\left[\left(P_0 - P(t)\right) + \left(R_0 - R(t)\right)\right]}{d(t)} - \bar{\tau}_R\bar{R}(t), \qquad \bar{R}(0) = \frac{2}{3}\bar{P}_0 \qquad (3)$$

$$\frac{dR(t)}{dt} = \bar{\tau}_R\bar{R}(t) - \left(\tau_R - \rho_R\right)R(t), \qquad R(0) = \frac{2}{3}P_0 \qquad (4)$$

$$\frac{da(t)}{dt} = a(t)\left[\theta - \zeta - \gamma C(t)\right], \qquad a(0) = a_0 \qquad (5)$$

$$\frac{dC(t)}{dt} = \lambda a(t)\left[\varepsilon I_C + \alpha C(t)\right]\left(\frac{P(t)}{P_0}\right)^{v} - \left(\tau_C - \rho_C\right)C(t), \qquad C(0) = C_0 \qquad (6)$$

where the influx-constraining function was

$$d(t) = \begin{cases} 1 & \text{if} \quad \ln\frac{a(t)}{a_0} < L \\ h\ln\frac{a(t)}{a_0} & \text{if} \quad \ln\frac{a(t)}{a_0} \geq L \end{cases} \qquad (7)$$

Here $I_P$ is the influx of $\bar{P}$ cells, i.e., the rate (all rates are in days$^{-1}$) of differentiation of $\bar{P}$ cells from stem cells, $\bar{\tau}_P$ is the rate of maturation of $\bar{P}$ cells into $P$ cells, and $\tau_P$ is the rate of natural death of $P$ cells; the quantities $\bar{\tau}_R$ and $\tau_R$ are defined in a fully analogical way. Further, $f$ is the amplifying coefficient of the linear feedback effect of $P$ and/or $R$ cell decrease on the influx of $\bar{P}$ and $\bar{R}$ cells at time $t$.

The quantity $\bar{c}_P a(t)C(t)$ is the rate of elimination of $\bar{P}$ cells due to the amount of HIV products $a(t)$ and the number of cytotoxic T cells $C(t)$ at time $t$. Analogously, $c_P a(t)C(t)$ is the rate of elimination of $P$ cells. The value $a_0$ is the function of the infectious dose of HIV, $\theta$ characterizes the growth rate of HIV, and $\gamma$ is the rate of inactivation of HIV products mediated by cytotoxic $C$ cells. The maturation of these cells from their precursors is assumed to be dependent on the encounter with HIV products and the effect of HIV specific helper T cells. $I_C$ is the influx of $C$ cell precursors, $\varepsilon$ their maturation rate, $\alpha$ the proliferation rate of $C$ cells under the antigenic stimulation by HIV products and helper T cell influence, and $\tau_C$ their natural death rate. Helper T cell effect on maturation and proliferation of $C$ cells is expressed by the ratio $P(t)/P_0$; the coefficient $v$ is introduced to characterize the intensity of this helper effect. The value $h$ characterizes HIV-constraining intensity on the $\bar{P}$ and $\bar{R}$ cell influx. Value $L$ defines the level, where such constraining (limiting) effect of $d(t)$ starts. Effects of therapeutic interventions are described by the following parameters: $\zeta$ - HIV elimination rate by AZT or passive immunization, $\lambda$ - immune response-enhancing factor, and $\rho_R$- and $\rho_C$-elimination rates of CD8+ and $C$ cells, respectively, by anti-CD8 antibodies.

If not otherwise stated, the model parameters in simulation runs were selected as follows: $\bar{\tau}_P = 0.2$, $\tau_P = 0.01$, $\bar{\tau}_R = 0.2$, $\tau_R = 0.01$, $\tau_C = 0.01$, $I_P = 1.0$, $I_C = 0.2$, $\bar{P}_0 = 5.0$, $P_0 = 100.0$, $\bar{R}_0 = 3.33$, $R_0 = 66.7$, $C_0 = 0.0$, $a_0 = 0.0005$, $f = 0.01$, $\alpha = 0.7$, $\varepsilon = 0.512$, $\gamma = 0.3$, $\theta = 0.02$, $v = 1.6$, $h = 3.5$, $L = 3.0$. Only mature CD4+ lymphocytes were assumed to be susceptible to HIV products, i.e. $\bar{c}_P = 0.0$, $c_P = 20.0$. As a rule, the parameter $e$ was used for final adjustment of the respective simulation run. If no therapeutic interventions are assumed ($\lambda = 1.0$, $\zeta = 0.0$, $\rho_R = 0.0$, $\rho_C = 0.0$), the resulting CD4+ standard curve characterizes best fit of the observed clinical data.
